# U-Net breast lesion segmentations for breast dynamic contrast-enhanced magnetic resonance imaging

**DOI:** 10.1117/1.JMI.10.6.064502

**Published:** 2023-11-20

**Authors:** Lindsay Douglas, Roma Bhattacharjee, Jordan Fuhrman, Karen Drukker, Qiyuan Hu, Alexandra Edwards, Deepa Sheth, Maryellen Giger

**Affiliations:** University of Chicago, Department of Radiology Committee on Medical Physics, Chicago, Illinois, United States

**Keywords:** breast lesion segmentation, fuzzy c-means, U-Net, breast magnetic resonance imaging, deep learning

## Abstract

**Purpose:**

Given the dependence of radiomic-based computer-aided diagnosis artificial intelligence on accurate lesion segmentation, we assessed the performances of 2D and 3D U-Nets in breast lesion segmentation on dynamic contrast-enhanced (DCE) magnetic resonance imaging (MRI) relative to fuzzy c-means (FCM) and radiologist segmentations.

**Approach:**

Using 994 unique breast lesions imaged with DCE-MRI, three segmentation algorithms (FCM clustering, 2D and 3D U-Net convolutional neural networks) were investigated. Center slice segmentations produced by FCM, 2D U-Net, and 3D U-Net were evaluated using radiologist segmentations as truth, and volumetric segmentations produced by 2D U-Net slices and 3D U-Net were compared using FCM as a surrogate reference standard. Fivefold cross-validation by lesion was conducted on the U-Nets; Dice similarity coefficient (DSC) and Hausdorff distance (HD) served as performance metrics. Segmentation performances were compared across different input image and lesion types.

**Results:**

2D U-Net outperformed 3D U-Net for center slice (DSC, HD p<0.001) and volume segmentations (DSC, HD p<0.001). 2D U-Net outperformed FCM in center slice segmentation (DSC p<0.001). The use of second postcontrast subtraction images showed greater performance than first postcontrast subtraction images using the 2D and 3D U-Net (DSC p<0.05). Additionally, mass segmentation outperformed nonmass segmentation from first and second postcontrast subtraction images using 2D and 3D U-Nets (DSC, HD p<0.001).

**Conclusions:**

Results suggest that 2D U-Net is promising in segmenting mass and nonmass enhancing breast lesions from first and second postcontrast subtraction MRIs and thus could be an effective alternative to FCM or 3D U-Net.

## 1 Introduction

Breast cancer is one of the leading causes of death in women. Magnetic resonance imaging (MRI) has had an integral role in improving breast cancer diagnoses and potentially reducing biopsies, in tumor staging, and in monitoring treatment response.^1^^–^^3^ Dynamic contrast-enhanced (DCE) MRI involves the acquisition of time series images after injection of a contrast agent; typically, one precontrast timepoint and multiple postcontrast timepoint images are acquired in 60 to 90 s intervals.^3^^,^^4^ Artificial intelligence methods, including detection, diagnosis, or segmentation tasks, have been developed to support radiologists in their interpretation decision-making process. The quality of segmentation needed, e.g., an approximate outline or a detailed contour, depends on the subsequent task.^5^ Precise lesion segmentation is required to extract relevant tumor features to be used in the classification components in computer-aided diagnosis (CADx) systems.^4^^,^^6^

A well-established and clinically used algorithm for breast lesion segmentation on DCE-MRI is a technique based on the fuzzy c-means (FCM) clustering algorithm, which analyzes the contrast uptake over time and yields volumetric segmentations.^4^ An alternative segmentation method is U-Net, a deep learning convolutional neural network, which produces segmentations based on a single timepoint.^7^ Without the requirement of using information from an entire dynamic time series, the U-Net has the potential to produce accurate segmentations from a variety of imaging sequences, including regular and abbreviated DCE-MRI acquisitions.^8^ The U-Net architecture can be designed to accept either 2D image slices or 3D image volumes.^9^ Several studies have been conducted to assess the performance of using 2D and 3D U-Nets for lesion segmentation from breast DCE-MRI.^10^^–^^14^ These methods have been developed using different DCE timepoints, datasets sizes, or unique ensembles of modified U-Nets. The evaluation criteria for these studies have been reported across a wide range, demonstrating the complexity of our task.^10^^–^^14^ The U-Nets used in our study were trained to segment masses and nonmass enhancing lesions from either first or second postcontrast subtraction images, i.e., subtraction images between the first or second postcontrast image and the precontrast image. Using the 2D U-Net, quasi-3D lesion segmentations can be obtained by stacking slice-by-slice segmentations; however, the lack of vertical (out-of-slice) continuity obtained by this “quasi-3D” U-Net may be a potential source of error that a fully 3D U-Net avoids.

In this study, we investigated the potential of using U-Nets in breast lesion segmentation on DCE-MRI by comparing the performances of 2D and 3D U-Nets relative to FCM.

## 2 Methods

The viability of using U-Nets in breast lesion segmentation on DCE-MRI was assessed by comparing the performances of 2D and 3D U-Nets in four evaluations. First, in comparison A, quasi-3D and 3D U-Nets were compared to FCM, which served as a surrogate reference standard.^15^ Second, in comparison B, the 2D U-Net, 3D U-Net, and FCM segmentations were compared to 2D radiologist delineations on lesion center slices for a subset of 71 lesions.^15^ Next, in comparison C, segmentations from first postcontrast subtraction images were compared to second postcontrast subtraction images for quasi-3D and 3D U-Nets. Finally, in comparison D, the segmentation performance of each method was evaluated for mass versus nonmass enhancing lesions.

### 2.1 Dataset

The dataset consisted of DCE-MRIs of 994 unique breast lesions (724 malignant and 270 benign) from 689 patients aged 23 to 89 years. The deidentified data were retrospectively collected at the University of Chicago over a span of 8 years (from 2005 to 2013) under Health Insurance Portability and Accountability Act-compliant Institutional Review Board protocols. Routine bilateral breast MRIs were acquired using a Philips Achieva scanner with either 1.5 T (N=473) or 3 T (N=216) magnet strength. The breast DCE-MRI protocol included a fat-saturated 3D T1 weighted spoiled gradient-echo sequence that was used to acquire precontrast and postcontrast images with a temporal resolution of 60 to 75 s (TE = 2.2 to 2.8 ms, TR = 4.5 to 7.5 ms, flip angle = 10 deg to 20 deg, in-plane resolution = 0.5 to 1.0 mm, FOV = 28.0 to 44.1 cm, matrix = 320 to 552×256 to 525, slice thickness = 1 to 3.5 mm, and interslice gap = 0.8 to 2.5 mm). Table 1 contains the clinical characteristics of the data obtained from pathology and radiology reports, including pathological truth (benign or malignant) and lesion type (mass or nonmass enhancement). A subset of 71 lesions was manually selected for radiologist delineations so that the distribution of pathological truth and lesion type within this subset was similar to the distribution in the overall group (Table 1). Table 2 presents size distributions of the lesions.

**Table 1:** Summary of the DCE-MRI dataset by lesion type. Lesions were categorized by pathological truth and enhancement type. Lesions that were not marked as either mass or nonmass enhancing were labeled “unknown.”

	Enhancement type	Pathological “truth”	
		Benign	Malignant
All lesions (N=994)	Mass	170	517
	Nonmass	49	175
	Unknown	51	32
Subset of lesions outlined by radiologist (N=71)	Mass	14	40
	Nonmass	7	10
	Unknown	0	0

**Table 2:** Summary of the DCE-MRI dataset by lesion size. Lesions were categorized by effective diameter (mm), defined by 2*√(A/π), where A is the area of the lesion in the center slice of the FCM segmentation in ${\mathrm{mm}}^{2}$.

	<5	5 to 9	10 to 14	15 to 19	>20
All lesions (N=994)	64	344	252	125	209
Subset of lesions outlined by radiologist (N=71)	2	11	32	15	11

### 2.2 Establishment of Reference Standards and Preprocessing

Each lesion had previously been segmented using a well-established, in-house, automated 3D FCM approach that yielded, as a surrogate “reference standard,” a 3D binary lesion segmentation.^4^ FCM segmentation was performed within a region defined by a human operator’s selection of a rectangular bounding box about the lesion in a middle slice along with an indication of the first and last slices in which the lesion appeared.^4^ The bounding-box volume of interest (VOI) for the FCM segmentation of each lesion was also used as input for subsequent U-Net segmentations of postcontrast subtraction images. Second postcontrast subtraction images were primarily used as inputs for the U-Net; however, first postcontrast subtraction images were introduced for evaluation in comparisons C and D of this study.

In addition, an expert radiologist (7 years of experience in breast imaging) manually delineated the lesion within the center slice of the second postcontrast subtraction VOI for the subset of 71 lesions. Here the radiologist segmentations were used as the “reference standard” for comparison B of this study. Since radiologist segmentations were only available for a limited set of center slices and FCM segmentations are used in an FDA-approved clinical breast MRI workstation,^3^ FCM segmentations served as a reasonable surrogate reference standard to train the U-Net architectures.

### 2.3 U-Net Architectures

Two different U-Net architectures were evaluated in this study. The first was a 2D U-Net.^7^ We found that the top and bottom slices of lesions were most difficult to segment, so those two slices were excluded from each lesion in training (though they remained in the test set lesions). The image slices of each lesion’s VOI were resized, by interpolation with a preserved pixel value range, to 256×256  pixels prior to input into the 2D U-Net. The 256×256  pixels probability map outputs, with values ranging from 0 to 1, were converted to binary segmentation images based on a threshold of 0.25. The 2D U-Net only processes one image slice at a time, so “quasi-3D” lesion segmentations were produced by stacking the 2D slice-by-slice segmentations obtained by the 2D U-Net to form a 3D volume. Hence, in this paper, “quasi-3D U-Net” refers to the volumetric segmentation produced by the 2D U-Net architecture.

The second architecture evaluated in this study was a 3D U-Net.^9^ This network is similar to the structure of the 2D U-Net, but it is modified with the added third dimension. Prior to input into the 3D U-Net, the lesion VOIs were resized, by interpolation with a preserved pixel value range, to 256×256×N  voxels (N is the number of slices in the lesion). The network produced 256×256×N  voxel probability map outputs, with values ranging from 0 to 1, which were converted to binary segmentation volumes based on a threshold of 0.23. The threshold for the binary conversion was selected from a range of values between 0.14 and 0.30 to produce the greatest mean Dice similarity coefficient (DSC) calculated from the resulting segmentations during training, hence the slight difference in threshold used for 2D U-Net and 3D U-Net.

### 2.4 Training and Statistical Analysis of Segmentation Performances

Fivefold cross-validation by lesion (N=994 lesions) was conducted to train and evaluate the U-Net models. The folds were partitioned such that each fold contained a similar distribution based on pathological truth (malignant or benign), lesion enhancement type, and lesion size. Additionally, since adjacent slices within the same lesion VOI often are very similar in appearance, all slices belonging to a given lesion were always allocated to the same fold. Training and test folds were allocated by lesion, i.e., not by slice or patient. The base U-Net models were trained using the Adam optimizer and a binary cross-entropy loss function; training was allowed to run for up to 200 epochs.

DSC and Hausdorff distance (HD) were used to evaluate the performances of the different segmentation methods relative to the specific reference standard.^16^^,^^17^ DSC is a measure of how well the areas of the two regions overlap, and HD is a measure of how well the margins of the two regions agree. Note that, throughout, better segmentation performance is indicated by higher DSCs and lower HDs. Predictions from the quasi-3D and 3D U-Nets were resized to their original lesion VOI dimensions before DSCs and HDs were calculated between the predictions and the reference standards. HDs were calculated for each slice, and the median HD for each lesion was used for 3D performance comparisons. To assess statistical significance of difference in performance, the Wilcoxon signed-rank test was used for matched cases in comparisons A, B, and C, and the Mann–Whitney U-test was used in comparison D due the analysis of unmatched cases.^16^^–^^19^ The Bonferroni correction was used to correct p-values for multiple comparisons in comparisons B, C, and D.^20^

### 2.5 Comparison A: Comparing Quasi-3D U-Net to 3D U-Net Using FCM as the Surrogate Reference Standard

**Fig. 1:**
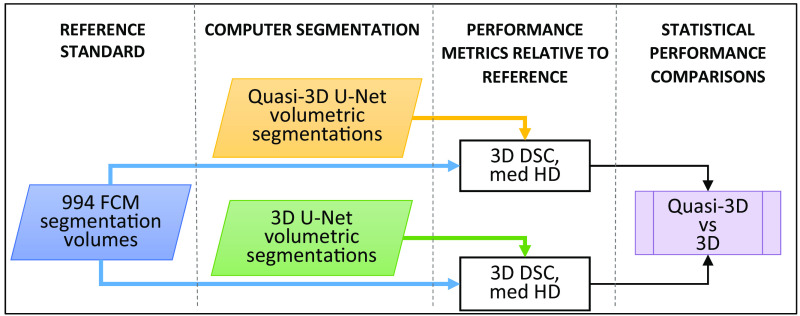
Flowchart of comparison A of this study (N=994). FCM lesion segmentation volumes were used as reference standard to compare quasi-3D U-Net (2D architecture) and 3D U-Net segmentations in a by-lesion fivefold cross-validation process. DSC, Dice similarity coefficient and med HD, median Hausdorff distance of all slices in the lesion. Wilcoxon signed-rank tests were performed on the resulting paired data.

The volumetric segmentations from quasi-3D and 3D U-Nets were compared and as previously noted, FCM segmentations served as the surrogate reference standard for the 994 lesions (Table 1). The Wilcoxon signed-rank test was used to assess statistically significant differences between quasi-3D and 3D U-Net segmentation performances (Fig. 1).

### 2.6 Comparison B: Comparing FCM, Quasi-3D U-Net, and 3D U-Net Using Radiologist-Delineations as the Reference Standard

**Fig. 2:**
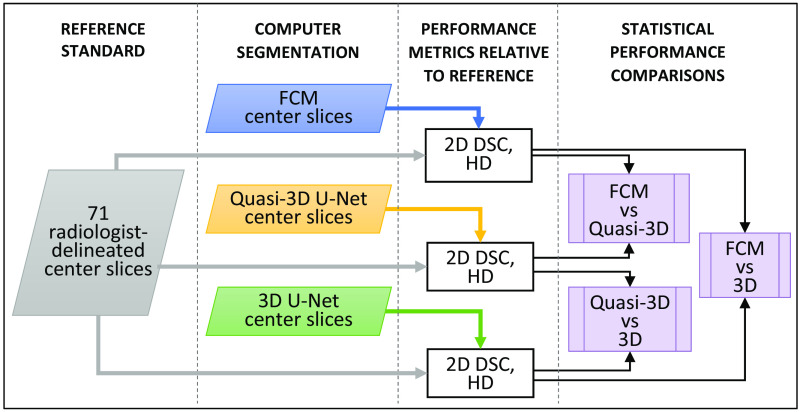
Flowchart of comparison B of this study (N=71). Radiologist segmentations were used as reference standard to compare FCM, quasi-3D U-Net, and 3D U-Net center slice segmentations. DSC, Dice similarity coefficient and HD, Hausdorff distance. Wilcoxon signed-rank tests were performed on the resulting paired data.

Next, FCM, quasi-3D U-Net, and 3D U-Net center slice segmentations were compared using the radiologist references available for the subset of 71 lesions. For each of the three segmentation methods, DSCs and HDs were calculated on the center slice with respect to the radiologist reference. Statistically significant differences between quasi-3D U-Net, 3D U-Net, and FCM segmentations were assessed using the Wilcoxon signed-rank test including a Bonferroni correction (Fig. 2).

### 2.7 Comparison C: Comparing Segmentation Across Postcontrast Timepoints (First Versus Second Postcontrast)

The segmentations obtained in comparison A using second postcontrast subtraction images as input were compared to those using the first postcontrast subtraction images for the quasi-3D and 3D U-Nets. Wilcoxon signed-rank tests were used to assess statistical significance between the results after a Bonferroni correction.

### 2.8 Comparison D: Comparing Segmentation Across Lesion Enhancement Types (Mass Versus Nonmass Enhancement)

Finally, the segmentation performances on mass and nonmass enhancing lesions were compared. The segmentations resulting from the first and second postcontrast subtraction inputs to the quasi-3D and 3D U-Nets evaluated in comparison C were compared based on lesion enhancement type. For each comparison, a Mann–Whitney U-test including a Bonferroni correction for statistical significance was used to compare the segmentation performances of the set of mass lesions to the set of nonmass lesions.

## 3 Results

### 3.1 Comparison A: Comparing Quasi-3D U-Net to 3D U-Net Using FCM as the Surrogate Reference Standard

Segmentation performance was assessed by comparing the medians of DSC and HD (Table 3). Note that better segmentation performance is indicated by higher DSCs and lower HDs. Of the 994 lesions in the dataset, the 3D U-Net failed to segment 6 lesions (from 3 unique patients) that were <9.1  mm in effective diameter and had an unknown enhancement type. Without prediction volumes available to compare to the reference standard, DSC was essentially zero and it was impossible to calculate HDs, therefore, these lesions were excluded from HD statistical comparisons for the 3D U-Net. The results of the Wilcoxon signed-rank test show that the quasi-3D U-Net statistically significantly outperformed the 3D U-Net in terms of DSC (p<0.001) and HD (p<0.001) for lesion segmentation from second postcontrast subtraction VOIs.

**Table 3:** Comparison A: summary statistics of the performance metrics of quasi-3D and 3D U-Nets as compared to FCM reference standards for volume segmentation. U-Nets were trained and tested using fivefold cross validation by lesion. Minimum, maximum, and median values of DSC and HD metrics of all cases are shown. Parenthetical values represent 95% confidence intervals.

Segmentation method	Min DSC	Max DSC	Median DSC	Min HD (mm)	Max HD (mm)	Median HD (mm)
Quasi-3D U-Net	0.270	0.955	0.780^a^ (0.774, 0.787)	0.737	73.6	7.30^a^ (6.79, 7.72)
3D U-Net	0^b^	0.935^b^	0.721^b^ (0.710, 0.732)	0.741^c^	98.2^c^	7.53^c^ (1.48, 56.3)

Note: DSC, Dice similarity coefficient and HD, Hausdorff distance (N=994).

a Statistically significantly greater performance after Bonferroni correction for two comparisons.

b Excluding six lesions with DSC = 0, minimum DSC = 0.035, maximum DSC = 0.935, and median DSC = 0.721 (0.710, 0.733).

c Due to failed segmentation, six lesions were excluded from 3D U-Net HD results because HD could not be calculated for those lesions.

### 3.2 Comparison B: Comparing FCM, Quasi-3D U-Net, and 3D U-Net Using Radiologist-Delineations as the Reference Standard

Based on the segmentation results for the subset of 71 lesions, we found that the center slices from each lesion segmentation produced by FCM, quasi-3D U-Net, and 3D U-Net had good agreement with the radiologist-segmented reference standard (Table 4). The statistical comparisons of performance between each segmentation method’s agreement with the reference standard is shown in Table 5. The results indicate that quasi-3D U-Net outperformed both 3D U-Net and FCM for lesion segmentation on second postcontrast subtraction center slices.

**Table 4:** Comparison B: summary statistics of the performance metrics of FCM, quasi-3D U-Net, and 3D U-Net, as compared to radiologist reference standard for center slice segmentation. U-Nets were trained and tested using fivefold cross validation by lesion. Minimum, maximum, and median DSC and HD metrics of all cases are shown. Parenthetical values represent 95% confidence intervals (N=71).

Segmentation method	Min DSC	Max DSC	Median DSC	Min HD (mm)	Max HD	Median HD (mm)
FCM	0.209	0.961	0.832 (0.813, 0.859)	0.796	31.2	4.06 (3.38, 4.80)
Quasi-3D U-Net	0.274	0.959	0.864 (0.845, 0.889)	0.796	28.5	3.28 (2.97, 4.50)
3D U-Net	0.246	0.952	0.802 (0.766, 0.834)	1.13	26.6	4.17 (3.04, 5.30)

Note: DSC, Dice similarity coefficient and HD, Hausdorff distance.

**Table 5:** Comparison B: statistical comparisons between the median performance metrics in Table 4 from FCM, quasi-3D U-Net, and 3D U-Net center slice predictions using radiologist-delineations as the reference standard. U-Nets were trained and tested using fivefold cross validation by lesion. Raw, uncorrected p-values from the Wilcoxon signed-rank test are reported; statistical significance was assessed after correcting for three comparisons (N=71).

Segmentation comparison	DSC comparisons	HD comparisons
FCM versus quasi-3D U-Net	Quasi-3D U-Net outperformed FCM p<0.001	Quasi-3D U-Net outperformed FCM p<0.05
Quasi-3D U-Net versus 3D U-Net	Quasi-3D U-Net outperformed 3D U-Net p<0.001	Quasi-3D U-Net outperformed 3D U-Net p<0.05
FCM versus 3D U-Net	FCM outperformed 3D U-Net p<0.001	Failed to reach statistical significance. p=0.753

Note: DSC, Dice similarity coefficient and HD, Hausdorff distance.

**Fig. 3:**
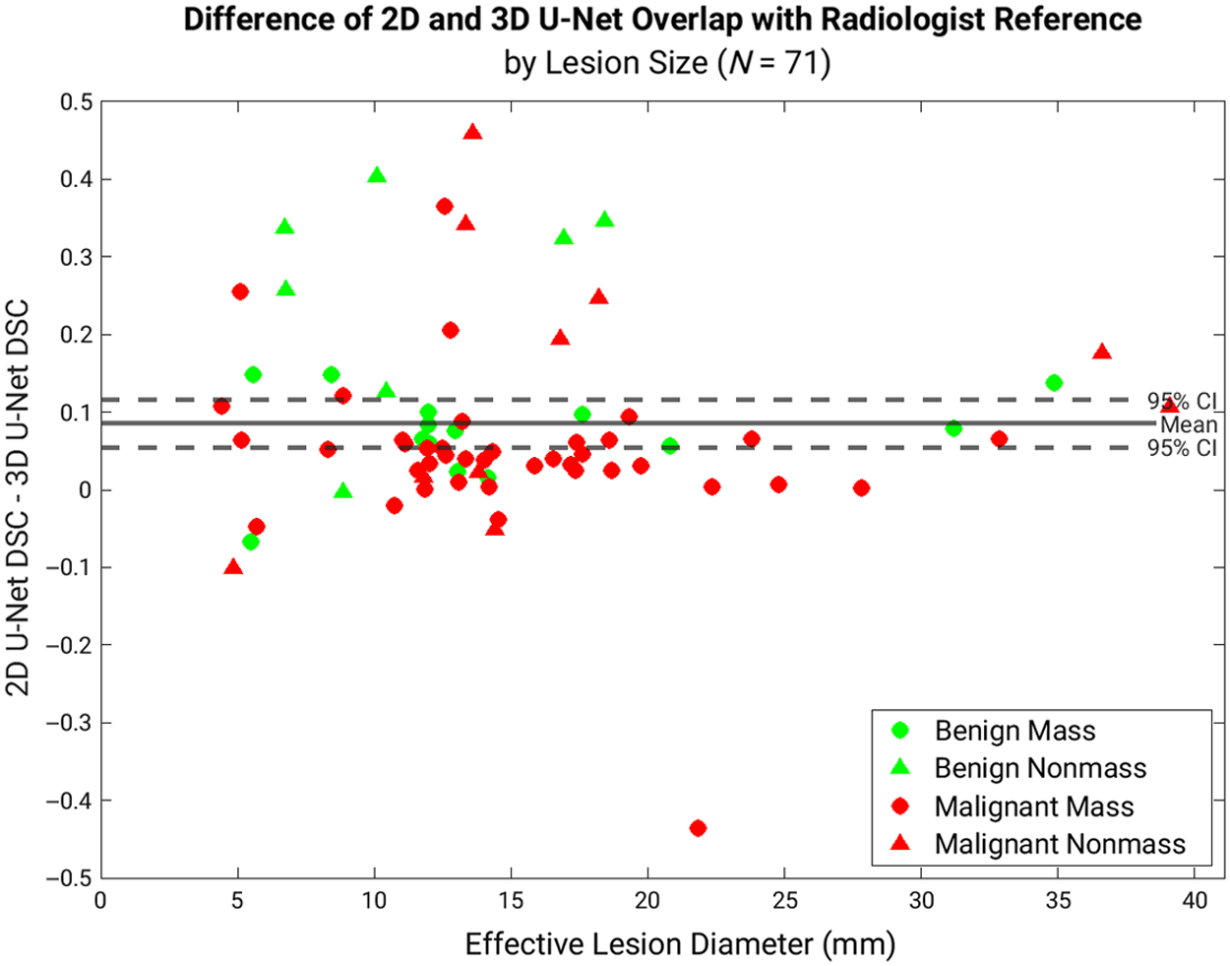
Difference in DSC calculated from the center slice of the quasi-3D (2D) U-Net or 3D U-Net and the radiologist reference versus lesion size. The majority of lesions yielded greater agreement between the radiologist and the quasi-3D (2D) U-Net than with the 3D U-Net. DSC, Dice similarity coefficient.

We observed improved U-Net segmentation agreement with the radiologist reference as lesion size increased, and Fig. 3 shows how quasi-3D U-Net yielded greater DSC values than 3D U-Net, relative to radiologist delineations, across all lesion sizes.

### 3.3 Comparison C: Comparing Segmentation Across Postcontrast Timepoints (First Versus Second Postcontrast)

**Fig. 4:**
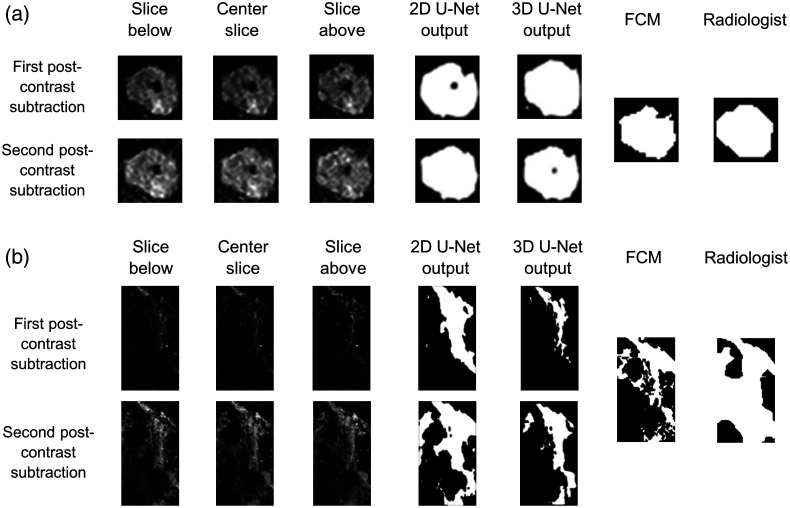
Example cases showing the center slice U-Net segmentations produced from the first or second postcontrast subtraction images for a (a) mass enhancing lesion and (b) nonmass enhancing lesion. The center slice FCM and radiologist references are also shown.

An example of the segmentations produced by the 2D U-Net, 3D U-Net, FCM, and radiologist for a mass and nonmass enhancing lesion is shown in Fig. 4. In the second postcontrast subtraction images, more lesion enhancement is provided to the U-Net, which as expected, tended to result in segmentations that more closely resembled FCM than segmentations from the first postcontrast subtraction segmentation inputs. Also as expected, the radiologist delineations acquired on the central slice of the second postcontrast subtraction image tended to resemble the center slice of the second postcontrast subtraction segmentation from the 2D U-Net.

The performance metrics calculated for the segmentations produced by the U-Nets from first and second postcontrast subtraction inputs are included in Table 6. The statistical comparisons between the resulting DSC and HD metrics for each method are shown in Tables 7 and 8. The results show statistically significantly greater performance from the second postcontrast subtraction inputs than from the first postcontrast subtraction inputs using the quasi-3D and 3D U-Nets, except in the case of nonmass enhancing lesions using the 3D U-Net. The results from both the first and second postcontrast subtraction inputs support the results found in comparisons A and B. The quasi-3D U-Net statistically significantly outperformed the 3D U-Net for the combined lesion types based on DSC, however HD failed to show statistically significant differences between quasi-3D and 3D U-Net for the first postcontrast subtraction input.

**Table 6:** Comparisons C and D: summary statistics of the performance metrics of quasi-3D U-Net and 3D U-Net, as compared to FCM surrogate reference standard. U-Nets were trained and tested using fivefold cross validation by lesion ($N_{\text{mass}}=687$ and $N_{\text{nonmass}}=224$).

Input	U-Net model	Lesion type	Median DSC	Median HD (mm)
First postcontrast subtraction images	Quasi-3D	Mass	0.7492	6.9375
		Nonmass	0.6126	12.0575
	3D	Mass	0.7357	6.6667
		Nonmass	0.5858	12.9417
Second postcontrast subtraction images	Quasi-3D	Mass	0.8059	6.8838
		Nonmass	0.6993	11.0459
	3D	Mass	0.7668	6.7734
		Nonmass	0.5458	15.1173

Note: DSC, Dice similarity coefficient and HD, Hausdorff distance.

**Table 7:** Comparisons C and D: statistical results for comparisons between input image type and lesion type using the Dice similarity coefficients of U-Net segmentations against FCM reference standard. U-Nets were trained and tested using fivefold cross validation by lesion. Raw, uncorrected p-values from the Wilcoxon signed-rank or Mann–Whitney U-test are reported. However, statistical significance was assessed after including a Bonferroni correction for four comparisons per sample ($N_{\text{mass}}=687$ and $N_{\text{nonmass}}=224$).

Input	U-Net	Lesions	First postcontrast subtraction images				Second postcontrast subtraction images			
			Quasi-3D		3D		Quasi-3D		3D	
			Mass	Nonmass	Mass	Nonmass	Mass	Nonmass	Mass	Nonmass
First postcontrast subtraction images	Quasi-3D	Mass	—	p<0.001	p<0.001	—	p<0.001	—	—	—
		Nonmass		—	—	p<0.001	—	p<0.001	—	—
	3D	Mass			—	p<0.001	—	—	p<0.001	—
		Nonmass				—	—	—	—	p<0.01
Second postcontrast subtraction images	Quasi-3D	Mass					—	p<0.001	p<0.001	—
		Nonmass						—	—	p<0.001
	3D	Mass							—	p<0.001
		Nonmass								—

**Table 8:** Comparisons C and D: statistical results for comparisons between input image type and lesion type using the Hausdorff distance of U-Net segmentations against FCM reference standard. U-Nets were trained and tested using fivefold cross validation by lesion. Raw, uncorrected p-values from the Wilcoxon signed-rank or Mann–Whitney U-test are reported. However, statistical significance was assessed after including a Bonferroni correction for four comparisons per sample ($N_{\text{mass}}=687$ and $N_{\text{nonmass}}=224$).

Input	U-Net	Lesions	First postcontrast subtraction images				Second postcontrast subtraction images			
			Quasi-3D		3D		Quasi-3D		3D	
			Mass	Nonmass	Mass	Nonmass	Mass	Nonmass	Mass	Nonmass
First postcontrast subtraction images	Quasi-3D	Mass	—	p<0.001	p<0.01	—	p<0.001	—	—	—
		Nonmass		—	—	p<0.001	—	p<0.001	—	—
	3D	Mass			—	p<0.001	—	—	p=0.298	—
		Nonmass				—	—	—	—	p<0.001
Second postcontrast subtraction images	Quasi-3D	Mass					—	p<0.001	p=0.212	—
		Nonmass						—	—	p<0.001
	3D	Mass							—	p<0.001
		Nonmass								—

### 3.4 Comparison D: Comparing Segmentation Across Lesion Enhancement Types (Mass Versus Nonmass Enhancement)

The results in Tables 6–8 demonstrate that, relative to the FCM reference standard, mass lesion segmentation statistically significantly outperformed nonmass enhancing lesion segmentation using first and second postcontrast subtraction image inputs to both the quasi-3D and 3D U-Nets. For nonmass enhancing lesions, quasi-3D U-Net always statistically significantly outperformed the 3D U-Net (as in comparisons A and B). For mass lesions, the DSC results indicate that quasi-3D U-Net statistically significantly outperformed the 3D U-Net (as in comparison B), however, the HD results from the first postcontrast subtraction inputs showed that the 3D U-Net statistically significantly outperformed quasi-3D U-Net.

## 4 Discussion

A crucial component of artificial intelligence systems is proper segmentation of lesions and other breast regions before subsequent extraction of quantitative values for clinically significant quantities. This study explored the performance of volumetric segmentations obtained with a 2D U-Net (quasi-3D U-Net) and a 3D U-Net. Segmentation performance was assessed against a well-established FCM method, which served as a surrogate reference standard, or radiologist reference segmentation.

There were several limitations of this study. First, the segmentation performances were evaluated within bounding-box VOIs; the inputs to the U-Net were based on the FCM volume dimensions. Future investigations could be focused on identifying lesions from the whole breast, rather than from a predefined region of interest. However, use of the bounding-box VOIs does mimic clinical practice where a radiologist may roughly indicate the region about a lesion as input to automatic characterization and CADx. Also there were a limited number of radiologist segmentations available, each acquired for the second postcontrast subtraction center slices (N=71 lesions); this could have influenced the results of the comparisons performed in comparison B. Additionally, there were no radiologist volume segmentations available for full lesion volumes, so FCM segmentations were used as surrogate reference standards. Finally, the 3D U-Net architecture may not be considered fully 3D since many lesions had too few slices to properly pool in the axial dimension. Future work may include an investigation of U-Net performance for breast lesion segmentation by exploring segmentation from abbreviated DCE-MRI sequences. Also, U-Nets may be trained with attention gating, which could potentially improve segmentation performance by focusing the network on the lesions and drawing attention away from the background tissue.

This study found that there were statistically significant differences in performance between U-Net and FCM segmentation methods, relative to each other and to a radiologist reference segmentation. In the task of segmenting breast lesions from second postcontrast subtraction DCE-MRI VOIs, the quasi-3D U-Net statistically significantly outperformed the 3D U-Net in segmenting volumes (N=988). Additionally, the comparison between center slices from FCM, quasi-3D U-Net, and 3D U-Net relative to the radiologist reference suggested that 2D U-Net outperforms FCM and 3D U-Net (N=71). Although the vertical (out-of-slice) context was an assumed advantage for the fully 3D U-Net, our results suggest that using the quasi-3D U-Net, which performs a series of 2D convolutions, max pooling, and upsampling operations, can accurately capture the lesion context and enable more precise localization of lesion pixels on a slice-by-slice basis. Another advantage of using the series of 2D convolutions, over 3D ones, is that less training is required due to the reduced complexity of the 2D net. Relative to FCM volumes, U-Net segmentations of second postcontrast subtraction inputs were statistically significantly greater than first postcontrast subtraction inputs, and segmentation of mass lesions statistically significantly outperformed nonmass lesion segmentation. Although improved segmentation using second postcontrast subtraction inputs were found, the 2D U-Net statistically significantly outperformed the 3D U-Net for the first postcontrast subtraction inputs; this could provide a potential benefit to abbreviated MRI applications. The results of this study suggest that using a 2D U-Net to yield quasi-3D U-Net segmentation of breast lesions from postcontrast subtraction DCE-MRIs is feasible and thus could be an effective alternative to more complex segmentation techniques. Ultimately, this work has the potential to encourage future incorporation of the quasi-3D U-Net method into artificial intelligence algorithms designed to improve the efficiency and efficacy of clinical workflows that include breast DCE-MRI.

## Data Availability

The data used for this manuscript, including DCE-MRIs and ROIs, are not publicly available due to patient privacy and data sharing agreements.
